# Prominin‐1‐expressing hepatic progenitor cells induce fibrogenesis in murine cholestatic liver injury

**DOI:** 10.14814/phy2.14508

**Published:** 2020-07-20

**Authors:** Michael Fenlon, Celia Short, Jiabo Xu, Nicolas Malkoff, Elaa Mahdi, Michelle Hough, Alison Glazier, Calvin Lee, Kinji Asahina, Kasper S. Wang

**Affiliations:** ^1^ Developmental Biology, Regenerative Medicine, and Stem Cell Program The Saban Research Institute Children’s Hospital of Los Angeles Los Angeles CA USA; ^2^ Southern California Research Center for ALPD & Cirrhosis Department of Pathology Keck School of Medicine University of Southern California Los Angeles CA USA

**Keywords:** Biliary atresia, cell lineage tracing, cholangiocyte, cholestasis, liver fibrosis, RNA‐seq

## Abstract

Cholestatic liver injury is associated with intrahepatic biliary fibrosis, which can progress to cirrhosis. Resident hepatic progenitor cells (HPCs) expressing Prominin‐1 (Prom1 or CD133) become activated and participate in the expansion of cholangiocytes known as the ductular reaction. Previously, we demonstrated that in biliary atresia, Prom1(+) HPCs are present within developing fibrosis and that null mutation of *Prom1* significantly abrogates fibrogenesis. Here, we hypothesized that these activated *Prom1*‐expressing HPCs promote fibrogenesis in cholestatic liver injury. Using *Prom1^CreERT2‐nLacZ/+^;Rosa26^Lsl‐GFP/+^* mice, we traced the fate of *Prom1*‐expressing HPCs in the growth of the neonatal and adult livers and in biliary fibrosis induced by bile duct ligation (BDL). *Prom1*‐expressing cell lineage labeling with Green Fluorescent Protein (GFP) on postnatal day 1 exhibited an expanded population as well as bipotent differentiation potential toward both hepatocytes and cholangiocytes at postnatal day 35. However, in the adult liver, they lost hepatocyte differentiation potential. Upon cholestatic liver injury, adult *Prom1*‐expressing HPCs gave rise to both PROM1(+) and PROM1(‐) cholangiocytes contributing to ductular reaction without hepatocyte or myofibroblast differentiation. RNA‐sequencing analysis of GFP(+) *Prom1*‐expressing HPC lineage revealed a persistent cholangiocyte phenotype and evidence of Transforming Growth Factor‐β pathway activation. When *Prom1*‐expressing cells were ablated with induced Diphtheria toxin in *Prom1^CreERT‐nLacZ/+^;Rosa26^DTA/+^* mice, we observed a decrease in ductular reactions and biliary fibrosis typically present in BDL as well as decreased expression of numerous fibrogenic gene markers. Our data indicate that *Prom1*‐expressing HPCs promote biliary fibrosis associated with activation of myofibroblasts in cholestatic liver injury.

## Graphical abstract

In this study, we demonstrate that during cholestatic liver injury following bile duct ligation that cholangiocytes derived from Prominin‐1‐expressing hepatic progenitor cells promote biliary fibrosis; ablation of Prom1‐expressing cells decreases fibrosis.

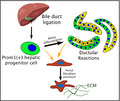

## 1 INTRODUCTION

Progression of liver fibrosis toward cirrhosis is the final common pathway for cholestatic liver injuries, such as biliary atresia (BA), progressive sclerosing cholangitis (PSC), and primary biliary cirrhosis (PBC) (Fabris, Spirli, Cadamuro, Fiorotto, & Strazzabosco, 2017). Accordingly, understanding liver fibrogenesis is critical for developing clinical interventions. During cholestatic liver injury, caused by impaired bile drainage from the liver, there is concurrent development of ductular reactions and adjacent periportal fibrosis. During initial stages of cholestasis, cholangiocytes actively undergo a limited number of rounds of mitosis to comprise the new cholangiocytes of evolving ductular reactions (Jors et al., 2015; Kamimoto et al., 2016; Rodrigo‐Torres et al., 2014). Cell lineage tracing studies indicate that hepatic progenitor cells (HPCs) residing in or adjacent to bile ductules within portal triads contribute the majority of newly differentiated cholangiocytes to ductular reactions (Miyajima, Tanaka, & Itoh, 2014; Sato et al., 2019). There is evidence that HPCs may also give rise to a small number of hepatocytes during cholestatic liver injury (Espanol‐Suner et al., 2012; Jors et al., 2015; Rodrigo‐Torres et al., 2014; Tarlow, Finegold, & Grompe, 2014).

HPCs reside in close proximity to portal fibroblasts, present within portal triads. Portal fibroblasts express Thymus cell antigen‐1 (Thy1) and Vimentin (Vim) and are distinct from hepatic stellate cells (HSCs) in that they do not store vitamin A lipids (Iwaisako et al., 2014; Katsumata, Miyajima, & Itoh, 2017; Lua, James, Wang, Wang, & Asahina, 2014; Lua et al., 2016; Wells, 2014). During early cholestatic injury, activated portal fibroblasts comprise the majority of myofibroblasts responsible for production of type I Collagen (Iwaisako et al., 2014). Associated with these myofibroblasts are HPCs and ductular reactions, which promote liver fibrosis in part via the conversion of latent pro‐fibrogenic Transforming Growth Factor‐β (TGF‐β) through cholangiocyte expression of Integrins, a large family of transmembrane heterodimers (Iwaisako et al., 2014; Peng et al., 2016).

We previously reported that expression of stem/progenitor cell marker *Prominin‐1* (*Prom1*), or CD133, by HPCs is upregulated in BA in regions of evolving intrahepatic biliary fibrosis (Mavila et al., 2014; Nguyen et al., 2017). Cell lineage tracing studies revealed that *Prom1*‐expressing HPCs contribute to hepatocytes in neonatal liver growth and to ductular reactions during liver injury (Zhu et al., 2016). Null mutation of *Prom1* leads to decreased ductular reaction and fibrosis in the neonatal murine model of BA. In infants with BA, there is a strong correlation between *PROM1* and genes associated with biliary fibrosis, such as biliary marker *KERATIN‐19* (*KRT19*) and Type I *COLLAGEN α1* (COL1A1) expression (Zagory et al., 2019). In this study, we hypothesize that the differentiation of *Prom1*‐expressing HPCs during cholestasis into a terminally differentiated cholangiocytes of ductular reactions is integral to the development of fibrosis. Herein, using transgenic mice for lineage tracing, transcriptomic, and loss‐of‐function analyses, we provide further evidence that PROM1 is integral to liver fibrogenesis during cholestatic liver injury.

## 2 MATERIALS AND METHODS

### 2.1 Animal experiments

*Prom1^CreERT2‐nLacZ^* knock‐in mice in C57BL/6N background were obtained from Dr. Richard Gilbertson (Zhu et al., 2016). *Rosa26‐stop‐enhanced green fluorescence protein* (*Rosa26^Lsl‐GFP^*, 006,148) and *Rosa26‐stop‐diphtheria toxin A* (*Rosa26^DTA^*, 009,669) mice in the C57BL/6J background were obtained from the Jackson laboratory (Bar Harbor, ME) (Srinivas et al., 2001; Wu, Wu, & Capecchi, 2006). For lineage tracing experiments, we generated *Prom1^CreERT2‐nLacZ/+^;Rosa26^Lsl‐GFP/+^* mice, heterozygous for each transgene, and injected tamoxifen (Sigma, St. Louis, MO, 200 µg/g body weight) intraperitoneally in neonatal mice on postnatal day (P) 1 and in 6‐week‐old adult mice (Zhu et al., 2016). Genotypes were confirmed by PCR prior to each experiment. To deplete *Prom1*‐expressing HPCs, we generated *Prom1^CreERT2‐nLacZ/+^;Rosa26^DTA/+^* mice and treated them with tamoxifen as above. Bile duct ligation (BDL) was performed by double ligation of the common bile duct with 3% isoflurane anesthesia under sterile conditions. Included were both male and female mice. Mice were given *ad libitum* chow, kept in clean conditions, with 12‐hr light and dark cycles. Prior to collection of tissue, mice were euthanized via CO_2_ asphyxiation followed by performing cervical dislocation. All animal experiments were conducted under a protocol approved by the Children's Hospital Los Angeles Institutional Animal Care and Use Committee.

### 2.2 LacZ, Sirius red, and immunofluorescence imaging

After mice were euthanized, livers were flushed with 1X phosphate‐buffered saline (PBS) via cardiac cannulation until the effluent from right ventriculotomies was clear. Livers were collected and fixed overnight with 4% paraformaldehyde in PBS (Wako Chemicals, Richmond, VA). Livers were then incubated with 30% sucrose in PBS overnight, and embedded in Tissue Tek O.C.T. compound (Sakura Fineteck, Torrance, CA) prior to −80°C freezer storage. Cryosections (10 µm) were used for LacZ staining as previously described (Berg et al., 2007) and immunofluorescence staining. Paraffin sections (5 µm) were used for Sirius red staining. LacZ, Sirius Red, and immunofluorescence staining were performed as previously described (Zagory et al., 2019). After LacZ staining, the sections were counterstained with hematoxylin. Primary antibodies used for immunofluorescence staining were KRT19 (100‐fold dilution, Dr. Joshua Friedman, University of Pennsylvania) and THY1 (100‐fold dilution, BD Pharmingen). Anti‐rabbit or rat secondary antibodies conjugated with Cy3 or FITC (200‐fold dilution, Jackson ImmunoResearch Labs) were used for detection of the primary antibodies. Nuclei were counterstained with DAPI. Images were acquired with a Leica DM5500B microscope and were processed with the Leica Suite Advanced Fluorescence 6,000 software (Leica Microsystems, Wetzlar, Germany). ImageJ (National Institutes of Health, Bethesda, MD) software was used to quantify cell positivity for LacZ and immunofluorescence staining. Cell counts were obtained from all animals in each condition (*n* = 5–8), three sections from each animal and five to eight images/animal were analyzed. Images were randomly selected and were excluded if did not capture periportal area for analysis.

### 2.3 Determination of Fibrosis

Sirius Red staining was quantified densitometrically using ImageJ software to determine the extent of fibrosis by calculating collagen proportionate area (CPA) (Zagory et al., 2019). CPA analysis was completed by an independent histology reviewer blinded to sample conditions(*N* = 4–6 animals in each condition, three sections per animal with four to eight images/animal analyzed). Our area of interest was evaluation of the periportal region; thus, after random selection of high‐powered frames, selections were excluded if they did not capture the periportal area.

### 2.4 Serum Bilirubin

Whole mouse blood was obtained via direct cardiac puncture at time of euthanasia. Serum was separated from whole blood using BD microtainer tubes with Lithium Heparin (BD Bioscience, San Diego, CA). Total serum bilirubin was determined using Total Bilirubin Reagent Set and a Bilirubin Calibrator Set was used for standardization (Pointe Scientific, Canton, MI).

### 2.5 Three‐dimensional liver imaging

Confocal microscopy was used for deep tissue imaging. After mice were euthanized, livers were flushed with 1X PBS via cardiac cannulation. Liver lobes were then collected and fixed overnight with 4% paraformaldehyde in PBS. Livers were then incubated with 30% sucrose in PBS overnight, and embedded in Tissue Tek O.C.T. compound prior to −80°C freezer storage. Fixed frozen lobes in OCT were placed in 1X PBS for 5 min twice at room temperature. Optical tissue clearing was accomplished with a modified version (UB1) of a urea‐based amino‐sugar mixture, known as UbasM, comprised of 25% Meglumine, 25% Urea, 20% 1,3‐Dimethyl‐2‐imidazolidinone, 0.2% Triton‐X100, and 29.8% water (Chen et al., 2017). Whole lobes were placed in 20 ml UB1 at 37°C with continuous rocking for 24 hr. After optical clearing, GFP fluorescence signals from samples were imaged on a Zeiss LSM 710 instrument. Z‐stacks were rendered and analyzed using Vision 4D image processing software (Arivis®, Munich, Germany).

### 2.6 Fluorescence‐activated cell sorting (FACS).

To analyze expression of GFP and PROM1, we minced the liver tissue and digested with PBS containing 0.1% collagenase (Sigma), 0.1% pronase (Sigma), and 0.01% DNase (Sigma) for 45 min at 37°C. After addition of FBS (10% final concentration), the dissociated cells were strained through a 70‐µm pore filter and red blood cells were removed with RBC lysis buffer (BD, Franklin Lakes, NJ). The resultant cells then underwent serial centrifugation to enrich the nonparenchymal fraction (Rountree, Ding, He, & Stiles, 2009). Cells were incubated with antibodies against PROM1 conjugated with PE (ThermoFisher Scientific, Waltham, MA) and propidium iodide and were analyzed by FACS Aria I (BD) at the CHLA FACS Core. After exclusion of dead cells stained with propidium iodide, expression of PROM1 and GFP was analyzed. Negative gating was set with simultaneously digested wild type livers. For RNA‐seq analysis, we sorted 1x10^5^ GFP(+) cells directly into Trizol solution (ThermoFisher Scientific).

### 2.7 RNA‐seq Analysis

Total RNA was purified using Trizol solution. RNA was further purified with silica bead Microprep columns (Qiagen, Germantown, MD). RNA quality was analyzed with an RNA Bioanalyzer (Agilent, Santa Clara, CA). Libraries were prepared with a Truseq mRNA library prep and sequencing was performed on an Illumina Nextseq Mid‐Output flow cell (Illumina, San Diego, CA) at the CHLA sequencing core. Sequencing analysis was performed with Partek Flow Genomic Analysis software (St. Louis, MO). Raw reads were trimmed for a minimum quality read of 20, with a minimum l of 25 bp. Reads were aligned and quantified using the Star aligner 2.5.3a (Mm10 GENCODE, release M18). Normalization was performed with the upper quartile method, with at least 10 reads. Pathway analysis of RNA sequencing was carried out with IPA. Enriched gene cutoff included those genes with a fold change > 1.5 and *p*‐value < .05.

### 2.8 Quantitative PCR

After snap freezing whole livers, total RNA was isolated using silica column‐based methods (Qiagen). cDNA were created with iScript reverse transcriptase (Bio‐Rad, Hercules, CA). Intron spanning, gene‐specific primers in conjunction with a universal hydrolysis probe library #1‐165 (Roche, San Diego, CA) were used for quantitative PCR analysis on the LightCycler 480 system (RocheProbe number and primer sequences are *Acta2* (probe #9, 5’‐TGA CGC TGA AGT ATC CGA TAG A‐3’ and 5’‐CGA AGC TCG TTA TAG AAA GAG TGG‐3’), *Col1α1* (probe #19, 5’‐ACC TAA GGG TAC CGC TGG A‐3’ and 5’‐TCC AGC TTC TCC ATC TTT GC‐3’), *Krt19* (probe #63, 5’‐CCT CAG GGC AGT AAT TTC CTC‐3’ and 5’‐TGA CCT GGA GAT GCA GAT TG‐3’), *Itgβ6* (probe #31, 5’‐TCT AAG GCC AAG TGG CAA AC‐3’ and 5’‐TGC TTC TCC CTG TGC TTG TA‐3’), *Prom1* (probe #32, 5’‐CTG CGA TAG CAT CAG ACC AA‐3’ and 5’‐TAT CCA CTG ATG GGA GCT GA‐3’), *Thy1* (probe #79, 5’‐GAA AAC TGC GGG CTT CAG‐3’ and 5’‐CCA AGA GTT CCG ACT TGG AT‐3’), *Vim* (probe #79, 5’‐TGC GCC AGC AGT ATG AAA‐3’ and 5’‐GCC TCA GAG AGG TCA GCA AA‐3’), and *Gapdh* (probe #80, 5’‐TGT CCG TCG TGG ATC TGA C‐3’ and 5’‐CCT GCT TCA CCA CCT TCT TG‐3’). The ΔΔCt method was used to calculate relative gene expression using *Gapdh* as the housekeeping gene.

### 2.9 Statistical Analysis

Statistics were performed with Graphpad Prism Version 6.05 (San Diego, CA). Analysis of variance with post hoc Tukey, and Mann‐Whitney tests were performed where appropriate. A *p* < .05 was considered statistically significant.

## 3 RESULTS

### 3.1 Bi‐lineage differentiation potential of Prom1‐expressing HPCs in neonatal livers

**Figure 1:**
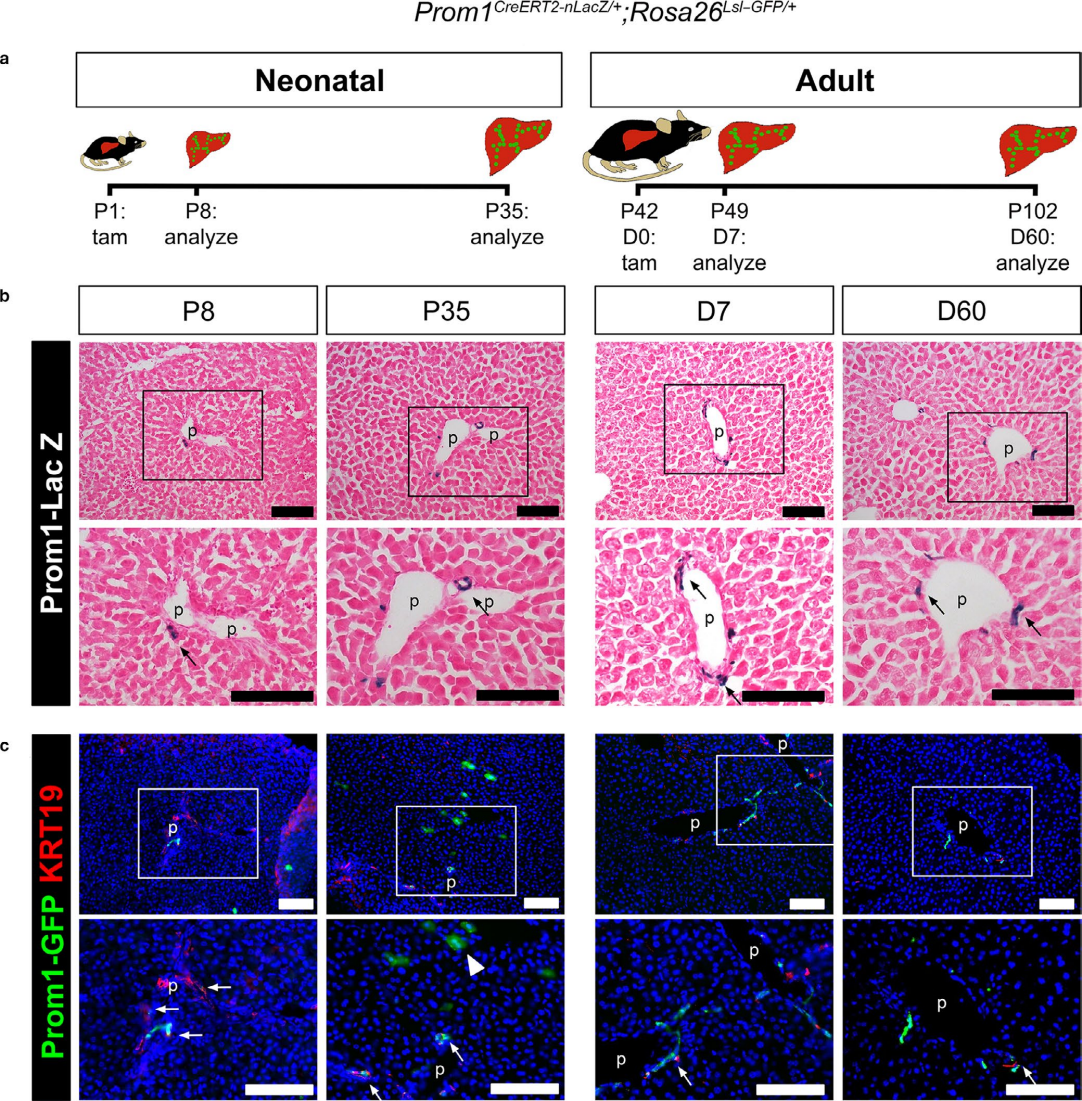
Loss of bi‐lineage differentiation potential of Prom1(+) HPCs in adult mouse liver. (a) Experimental design. In *Prom1^CreERT2‐nLacZ/+^;Rosa26^Lsl‐GFP/+^* mice, LacZ is expressed in the nuclei of cells expressing Prom1. Cells expressing Prom1 and their downstream progeny at the time of tamoxifen injection express GFP. Neonatal mice were injected with tamoxifen at P1 and their livers analyzed at P8 and P35 (*n* = 4 for each group, two males and two females). Six‐week old (P42) adult mice were injected with tamoxifen (D0) and their livers analyzed on day 7 and 60 (*n* = 4 for each group, two males and two females) There were no unexpected mortalities prior to experimental endpoints for all animals enrolled in this study. (b) LacZ staining of the neonatal livers (P8 and P35) or adult livers (D7 and D60) after tamoxifen injection. Arrows indicate some of the LacZ(+) cells (blue nuclei) in the bile duct adjacent to the portal vein (p). Sections were counterstained with hematoxylin. (c) Immunofluorescence staining of GFP and KRT19 in neonatal and adult livers after tamoxifen injection. Arrows indicate dual GFP(+) KRT19(+) HPCs in the bile duct. An arrowhead indicates GFP(+) KRT19(‐) hepatocytes in the neonatal liver at P35. Nuclei were counterstained with DAPI. Bar, 100 µm

To characterize cells expressing *Prom1* and trace their fate in the liver growth, we generated *Prom1^CreERT2‐nLacZ/+^;Rosa26^Lsl‐GFP/+^* mice, heterozygous for each transgene, where *Prom1*‐expressing cells express *CreERT2* and n*LacZ* (Figure 1a) (Zhu et al., 2016). Following tamoxifen injection to mice, *Prom1*‐expressing cells irreversibly express GFP. Using this mouse line, we expect to detect *Prom1*‐expressing cells as LacZ(+) cells and the progeny of *Prom1*‐expressing cells as GFP(+) cells with or without LacZ expression in the liver. To examine the fate of *Prom1*‐expressing HPCs in liver growth, we injected tamoxifen into neonatal mice on postnatal day (P) 1 and analyzed the liver on P8 and P35 (Figure 1a). We also injected tamoxifen into 6‐week‐old adult mice and analyzed the liver 7 and 60 days later. There were no mortalities prior to expected experimental endpoints. In both neonatal and adult livers, LacZ was expressed in equal number and exclusively located in a subset of cholangiocytes adjacent to the portal vein (Figure 1b). There was no LacZ expression detected outside of the periportal area or in hepatocytes. After tamoxifen injection at P1, neonatal livers at P8 showed the GFP expression only in KERATIN‐19 (KRT19)(+) cholangiocytes (Figure 1c). By P35, the GFP expression was observed in both KRT19(+) cholangiocytes and a few KRT19(‐) bi‐nucleated hepatocytes in the neonatal liver outside of the periportal area (Figure 1c). In contrast in the adult liver, tamoxifen‐labeled GFP(+) cells remained constant in number and within the bile ducts; there were no detectable GFP(+) hepatocytes by 60 days after treatment (Figure 1c). In both neonatal and adult livers at seven days post‐injection, LacZ staining and GFP expression were similar in number and location (Figure 1b,c), indicating *Prom1^CreERT2‐nLacZ/+^;Rosa26^Lsl‐GFP/+^* effectively drives recombination in this model. We confirmed no leakiness of the GFP reporter in the liver without tamoxifen treatment (data not shown). Our data indicate that *Prom1*‐expressing HPCs in the neonatal liver possess baseline generative capacity of bi‐lineage differentiation toward biliary epithelial cells and hepatocytes during the growth of the neonatal liver; in contrast, in the adult, the generative capacity of *Prom1*‐expressing HPCs is decreased. Given that Zhu *et al*. observed near complete GFP positivity in hepatocytes by P180 after P1 injection (Zhu et al., 2016), it is clear that *Prom1*‐expressing HPC generative capacity is arrested prior to 6 weeks of age and that further expansion of the GFP(+) hepatocytes subpopulations results from hepatocyte mitoses.

### 3.2 Prom1‐expressing HPCs possess only cholangiocyte differentiation following cholestatic liver injury.

**Figure 2:**
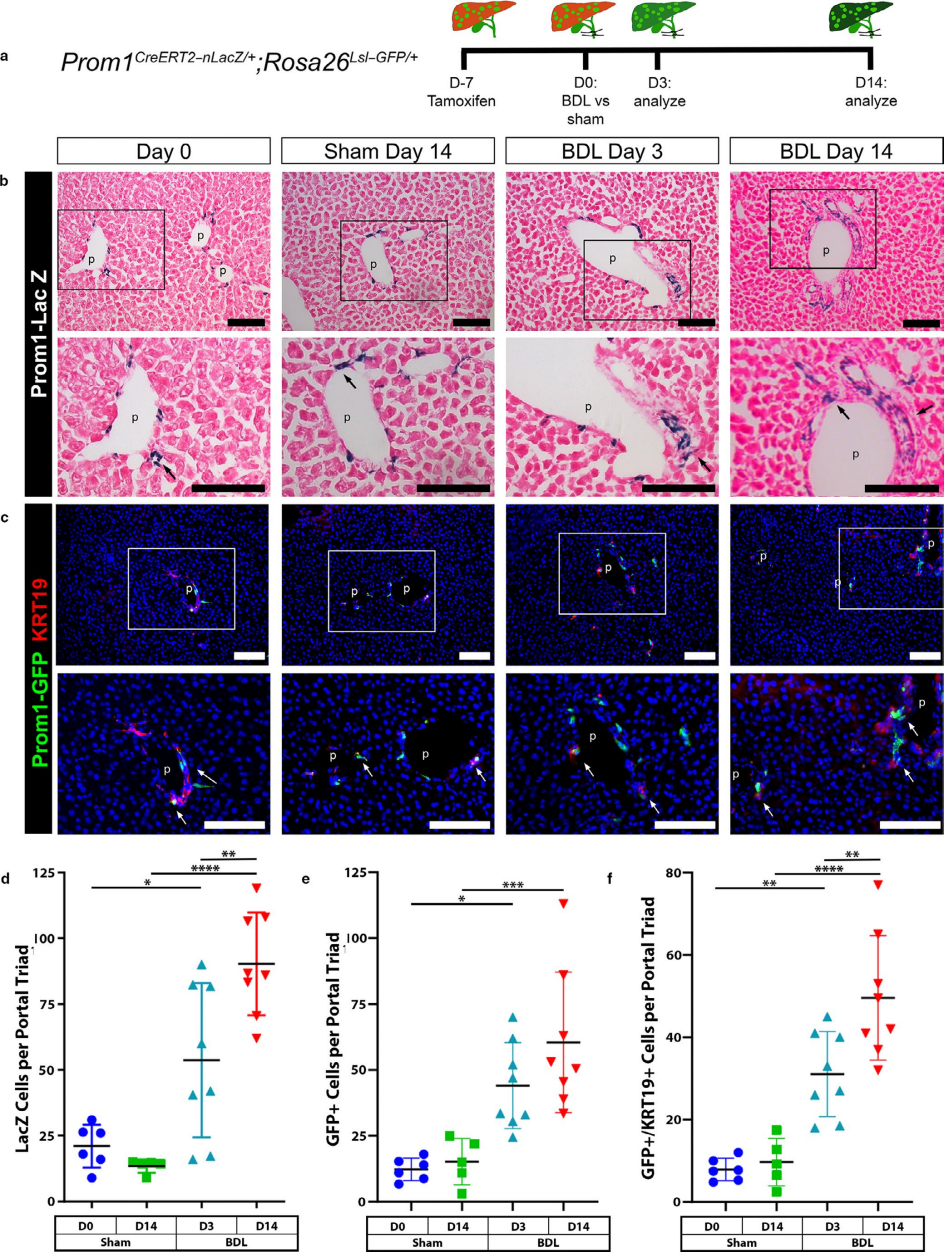
Differentiation of Prom1(+) HPCs to cholangiocytes in BDL‐induced biliary fibrosis. (a) Experimental design. Seven days after tamoxifen injection, 6‐week‐old *Prom1^CreERT2‐nLacZ/+^;Rosa26^Lsl‐GFP/+^* mice were subjected to a BDL or sham surgery. The liver tissues were collected from Sham D0 (*n* = 6, three males and three females), Sham D14 (*n* = 5, three males and two females), BDL D3 (*n* = 8, four males and four females), and BDL D14 (*n* = 8, 5 male and 3 female). Eight mice were excluded from analysis after being enrolled in the BDL surgery due to intraoperative death (*n* = 2) or early postoperative mortality prior to experimental endpoint (*n* = 6). There were no unexpected mortalities following sham surgery. (b) LacZ staining. Arrows indicate LacZ(+) HPCs in the bile duct adjacent to the portal vein (p). Sections were counterstained with hematoxylin. (c) Immunofluorescence staining of GFP and KRT19. Arrows indicate GFP(+) KRT1919(+) HPCs near the portal vein. Nuclei were counterstained with DAPI. Bar, 100 µm. (D‐F) Quantification of LacZ expressing cells per portal triad (d), GFP(+) cells per portal triad (e), and double GFP(+)/KRT19(+) cells per portal triad (f) at D0, D3, or D14 after BDL or sham surgery. * *p* < .05, ** *p* < .01, *** *p* < .001, and **** *p* < .0001

The intrahepatic biliary system changes their structure dynamically in cholestatic liver injury (Miyajima et al., 2014). To better characterize how *Prom1*‐expressing HPCs contribute to cholestatic liver injury, we labeled *Prom1*‐expressing cells by tamoxifen‐induced GFP in 6‐week‐old adult *Prom1^CreERT2‐nLacZ/+^;Rosa26^Lsl‐GFP/+^* mice. Seven days after tamoxifen injection, we performed BDL or sham surgery and traced the fate of *Prom1*‐expressing HPCs in cholestatic liver injury (Figure 2a). Seven days after tamoxifen injection at D0, LacZ and GFP expression was only observed in a subset of periportal cells as expected (Figure 2b,c). The number of LacZ(+) HPCs significantly increased after BDL, but not by sham operation (Figure 2d). Concordantly, GFP(+) cells were significantly increased by BDL (Figure 2e). These GFP + cells remained predominantly within the immediate periportal region and ductular reactions and were localized with or adjacent to KRT19(+) cells (Figure 2c). Double GFP(+) and KRT19(+) cells increased after BDL (Figure 2c,f). As expected, LacZ(+) and GFP(+) HPCs remained in the bile duct in the sham control group at D14.

**Figure 3:**
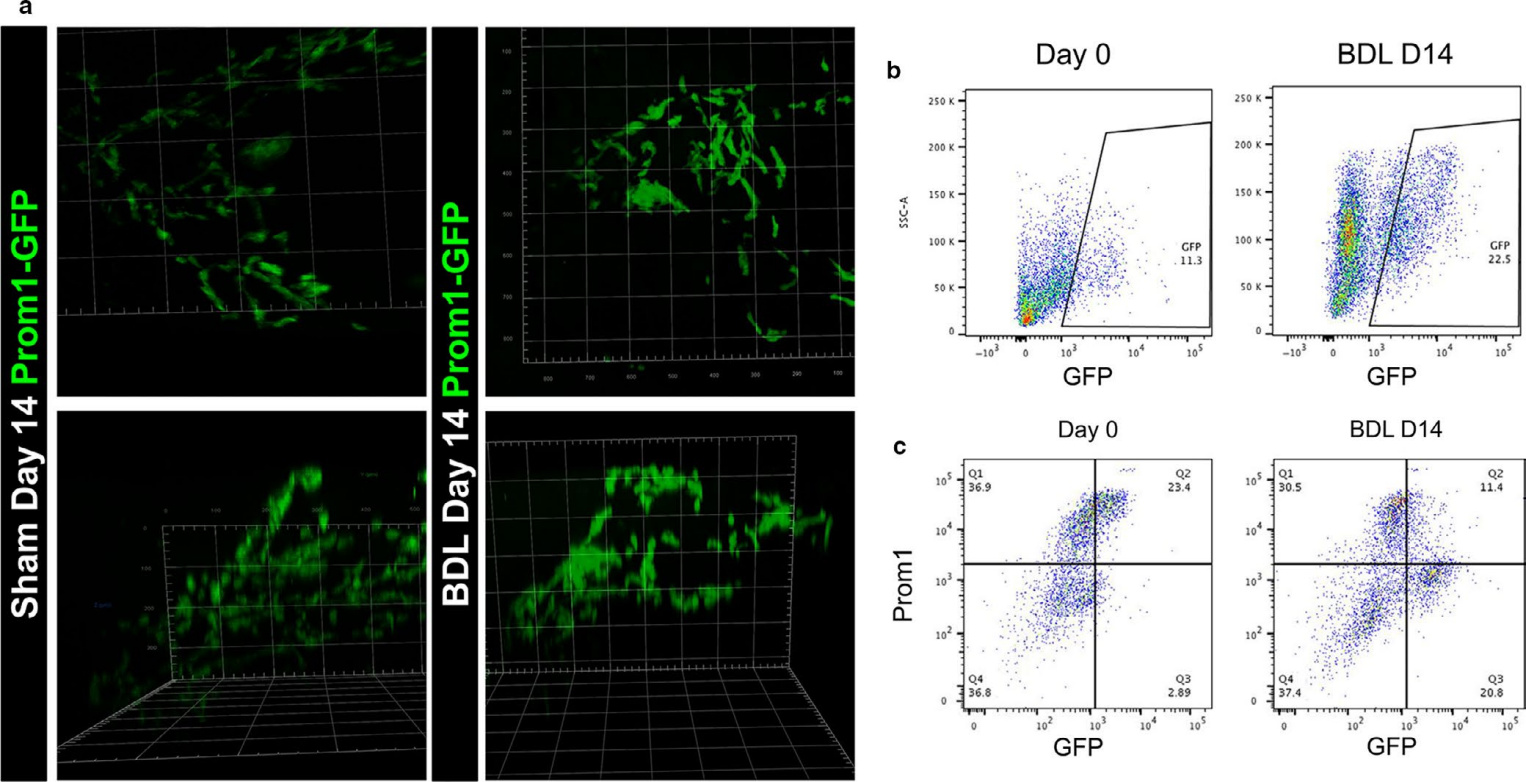
Differentiation of Prom1(+) HPCs to Prom(‐) cholangiocytes in BDL‐induced biliary fibrosis. (a) Three‐dimensional images of GFP(+) biliary trees in cleared sham control liver or fibrotic liver after BDL. (b) FACS of GFP expression in nonparenchymal cells prepared from *Prom1^CreERT2‐nLacZ/+^;Rosa26^Lsl‐GFP/+^* mouse livers. GFP(+) cells are gated. (c) FACS of GFP and PROM1 expression in the nonparenchymal cells. Note that GFP(+) PROM1(+) cells shift to GFP(+) PROM1(‐) cells by BDL

As the *Prom1*‐expressing HPC progeny reside within a continuous band of biliary cells following the arborizing portal vein, we opted to visualize the GFP lineage by whole mount, three‐dimensional confocal microscopy. To this end, we optically cleared liver lobes and detected the GFP expression with imaging z‐stacks. As shown in Figure 3a, there was an increase in the number and density of biliary trees composed of GFP(+) HPC progenies 14 days after BDL compared to sham. This can also be viewed in three‐dimensional video renderings with Supplemental video S1 (https://doi.org/10.6084/m9.figshare.11808990

URL: https://figshare.com/s/ec695354b2441a3f2373).

To further characterize *Prom1*‐expressing HPCs and their progenies, the non‐parenchymal fractions prepared from these liver tissues were analyzed by FACS. After tamoxifen injection, 11% of nonparenchymal cells expressed GFP at D0; this increased to 22% after BDL on d14 (Figure 3b). Before BDL, 89% of GFP(+) cells were co‐expressed PROM1 (Figure 3c). Fourteen days after BDL, the GFP(+) PROM1(+) population decreased to 35.4% with majority of GFP(+) cells being PROM1(‐) (Figure 3c). Our data indicate that the *Prom1*‐expressing HPC population expands in response to cholestatic liver injury and remains in the periportal and ductular reaction regions of injury. Furthermore, these data indicate that progeny of PROM1(+) HPCs lose expression of PROM1 as they differentiate into biliary epithelial cells during biliary fibrosis, without evidence of hepatocyte differentiation.

### 3.3 Transcriptomic changes of Prom1‐expressing HPCs in cholestatic liver injury

**Figure 4:**
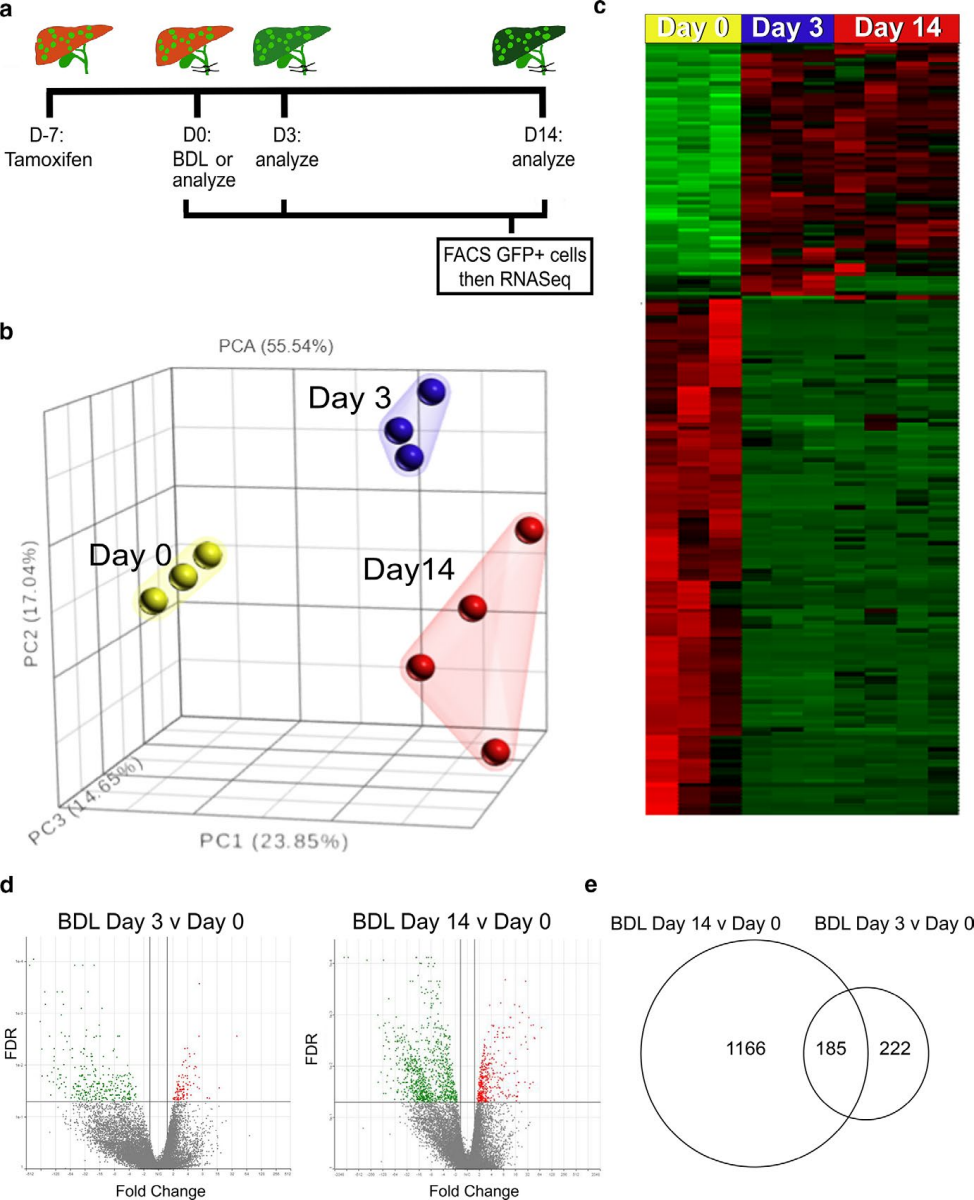
RNA‐seq analysis of GFP(+) HPCs and their progenies in normal and fibrotic livers. (a) GFP(+) cells were isolated from the 6‐week old *Prom1^CreERT2‐nLacZ/+^;Rosa26^Lsl‐GFP/+^* mouse livers before (day 0) and after BDL (day 3; day 14) by FACS. Total RNAs isolated from GFP(+) populations were subjected to RNA‐seq analysis. Eight mice were excluded from analysis after being enrolled in the BDL surgery due to intraoperative death (*n* = 3) or early postoperative mortality prior to experimental endpoint (*n* = 5). One sample from D0 and D3 post BDL was excluded from RNA seq due to poor RNA quality and two samples from D14 were excluded after RNA seq due to insufficient total reads after trimming and alignment. RNA seq analysis included D0 (*n* = 3, one male and two females), D3 (*n* = 3, two males and one female), and D14 (*n* = 4, three males and one female) (b) Low‐dimensional principal component analysis of each group. (c) Hierarchical clustering analysis of each group. (d) Volcano plots between BDL day 3 versus day 0 control (left) and BDL day 14 versus day 0 control (right). (e) Between BDL day 14 and day 0, there are 1,351 genes differentially expressed. Between BDL day 3 and day 0, there are 185 common genes to late differentiation

To further characterize genome‐wide transcriptional changes in Prom1 HPC differentiation, we isolated GFP(+) cells from *Prom1^CreERT2‐nLacZ/+^;Rosa26^Lsl‐GFP/+^* mouse livers treated with tamoxifen before (day 0, *n* = 3) and after BDL (day 3, *n* = 3; day 14, *n* = 4) and analyzed mRNA expression by RNA‐seq (Figure 4a). On low‐dimensional principal component analysis, biological replicates most closely associated with members from the same group (Figure 4b). Similarly, each time point aggregated on hierarchical clustering analysis (Figure 4c). With increasing duration of injury, variability increased, with BDL day 14 replicates more variable than Day 0 baseline HPCs. In early injury (BDL day 3 versus Day 0), there were 407 differentially expressed genes, with 105 upregulated and 302 downregulated (based on a False Discovery Rate (FDR) ≤ 0.05 and a fold change > 1.5) (Figure 4d). Between late injury on BDL day 14 and day 0, there were 1,352 genes differentially expressed, 958 genes were downregulated, while 394 were up‐regulated (Figure 4d). Early changes (between BDL day 3 and day 0) had 185 common genes as late differentiation (BDL day 14 versus day 0) (Figure 4e).

### 3.4 Prom1‐expressing HPC progeny retains a cholangiocyte phenotype with concurrent activation of TGF‐β signaling

**Figure 5:**
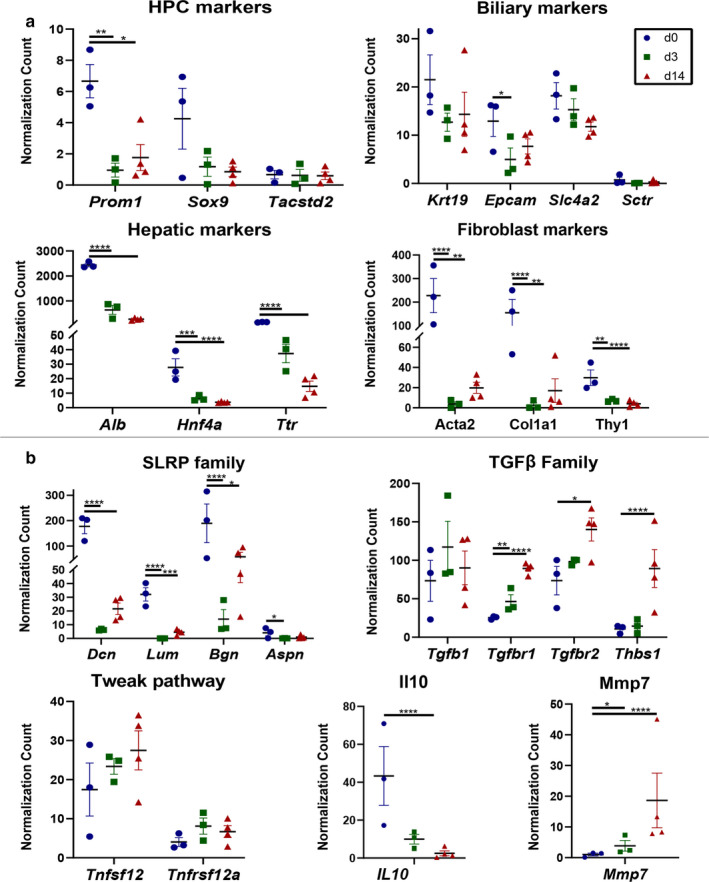
Expression of marker genes in different GFP populations in biliary fibrosis. Expression profiles identified by RNA‐seq analysis of markers for HPCs, cholangiocytes, hepatocytes, and mesenchymal cells (a), as well as genes from the SLRP family, TGF‐β family, TWEAK family, *Il10*, and *Mmp7* (b). *p < .05, **p < .01, ***p < .001, and ****p < .0001

We next analyzed genes highlighted in the RNA‐seq data from GFP(+) *Prom1*‐expressing HPC lineage for gene markers of particular cell phenotypes comparing days 0, 3, and 14 post‐injury (Table 1). Following BDL, there was a significant decrease in *Prom1* expression within the HPC lineage population (Figure 5a), consistent with our observations by FACS (Figure 3c). In terms of other putative HPC markers, *Sox9* demonstrated a trend toward a decrease in GFP(+) cells after BDL while *Tacstd2* (also known as Trop2) was unchanged (Figure 5a). Expression of genes encoding biliary epithelial markers *Krt19*, biliary anion transporter *Slc4a2* (*Ae2*), and *Secretin Receptor*, *Sctr*, likewise were unchanged (Figure 5a). There was a transient decrease in biliary gene marker *Epcam* at d3 which rebounded by d7. There was decreased expression of genes encoding hepatocyte markers *Albumin* (*Alb*), *Hnf4α*, and *Transthyretin* (*Ttr)* (Figure 5a). Finally, we observed decreased expression of genes encoding activated portal fibroblastic markers *α‐Smooth muscle actin* (*Acta2*), *Collagen‐1α1* (*Col1a1*), and *Thymus cell antigen‐1* (*Thy1*) in the GFP(+) *Prom1*‐expressing HPC lineage. These data collectively indicate that during evolving cholestatic liver injury following BDL, *Prom1*‐expressing HPCs retain their biliary epithelial phenotype while losing *Prom1* expression. Moreover, *Prom1*‐expressing progenitors do not appear to give rise significantly to hepatocytes or activated portal fibroblasts following BDL.

**Table 1:** list of top 20 up and down regulated genes at d3 and d14 versus d0, sorted by false discovery rate and arranged by ascending/descending fold change

Days 3 versus Day 0				Days 14 versus Day 0				
Up regulated		Down regulated		Up regulated			Down regulated	
Gene ID	Fold Change	Gene	Fold Change	Gene	Fold Change		Gene	Fold Change
Mmp12	39.82	Lum	−440.57	Gpnmb		85.31	Fabp1	−1359.03
Trem2	6.84	Dpt	−363.68	Chil3		58.14	Mup22	−921.22
Txnrd1	6.82	Mfap4	−215.21	F13a1		33.29	Cd209f	−103.21
Vat 1	6.73	Sfrp1	−209.92	Syngr1		33.08	Cxcl13	−52.02
Ccl6	5.86	Mylk	−119.52	Mcub		26.20	Hspa1b	−26.63
Nce1	5.58	Sparcl1	−98.18	Cx3cr1		24.40	C6	−25.13
Gla	5.37	Col1a2	−89.50	Myo5a		23.90	C2	−24.97
Lgals3	4.36	Myl9	−52.73	Ccr1		17.28	Clec4f	−24.87
Tmem37	4.26	Tagln	−42.48	Fabp5		16.43	Hmgcs2	−24.48
Rab7b	4.09	Tpm2	−38.42	Trem2		14.41	Serpina1c	−24.09
Cd3001f	3.91	Fm02	−30.82	Cyp4f18		13.59	Timd4	−22.15
Gusb	3.86	Dcn	−27.93	Emb		12.96	Hspala	−20.91
Tspan4	3.83	Rgs5	−21.02	Emp1		12.68	Serpina1d	−13.69
Pmp22	3.78	Mgp	−19.03	Tgfbi		11.15	Adh1	−12.55
Creg1	3.64	Serpinh1	−16.52	Cd63		9.97	Serpina1a	−12.23
Htatip2	3.57	Col3a1	−15.89	Arl11		9.45	Apoc1	−10.97
Gsr	3.53	Col14a1	−15.89	Gla		7.63	Aldh1a1	−10.20
Sgpl1	3.53	Cavin1	−14.86	Nech1		5.29	Ly6a	−7.61
Ephx1	3.43	Hmgcs2	−8.51	Hexb		5.10	Aldob	−7.48
Colgalt1	3.06	Tpm1	−6.87	Tlr13		4.5	IrF7	−7.32

Ingenuity pathway analysis (IPA) was also performed on the differentially expressed genes of the GFP(+) *Prom1*‐expressing HPC lineage. As noted above, the majority of significantly differentiated genes are downregulated. Notably, on evaluation of the most downregulated genes at day 3 and day 14 time points, several were members of the small leucine‐rich proteoglycan (SLRP) family, including *Decorin* (*Dcn*), *Biglycan* (*Bgn*), *Lumican* (*Lum*), and *Asporin* (*Aspn*) (Figure 5b). This family is known to inhibit the bioactivity of TGF‐β signaling pathways (Costanza, Umelo, Bellier, Castronovo, & Turtoi, 2017). Additionally, IPA analysis revealed trends of *Tgfb1* toward increased expression on day 3 but was overall constant, while *Tgfbr1* and *Tgfbr2* were both significantly upregulated by day 14 (Figure 5b). Integrins and Thrombospondin 1 (Thbs1) are known to release active TGF‐β ligands (Murphy‐Ullrich & Suto, 2018; Nishimura, 2009). Expression of *Thbs1* was upregulated in GFP(+) cells at d14 (Figure 5b), while expression of *Itgav*, *Itgb5*, and, *Itgb6* remained constant (data not shown). TNF‐like weak inducer of apoptosis, TNFSF12 (also known as TWEAK), and its receptor, Fn14 (TNFRSF12a), are downstream targets of the TGF‐β signaling ( Chen et al., 2018) and both trend toward upregulation, however, do not reach significance (Figure 5b). The RNA‐seq analysis suggests that Prom1‐expressing HPC progeny is involved in activation of TGF‐β signaling in biliary fibrosis.

Additional areas of interest in our IPA included the Notch signaling pathway, as it has been implicated in biliary epithelial cell differentiation after liver injury (Boulter et al., 2012; Zhang et al., 2016), our sequencing data were consistent with increased expression of *Notch2* (data not shown). GFP(+) HPCs downregulated *Il10*, an immunosuppressive cytokine (Figure 5b). Expression of *Mmp7* was also significantly upregulated at D14 (Figure 5b), MMP7 has been evaluated as potential BA marker with significant expression seen in BA infants (Lertudomphonwanit et al., 2017; Yang et al., 2018).

### 3.5 Ablation of Prom1‐expressing HPC mitigates fibrogenic response of BDL

**Figure 6:**
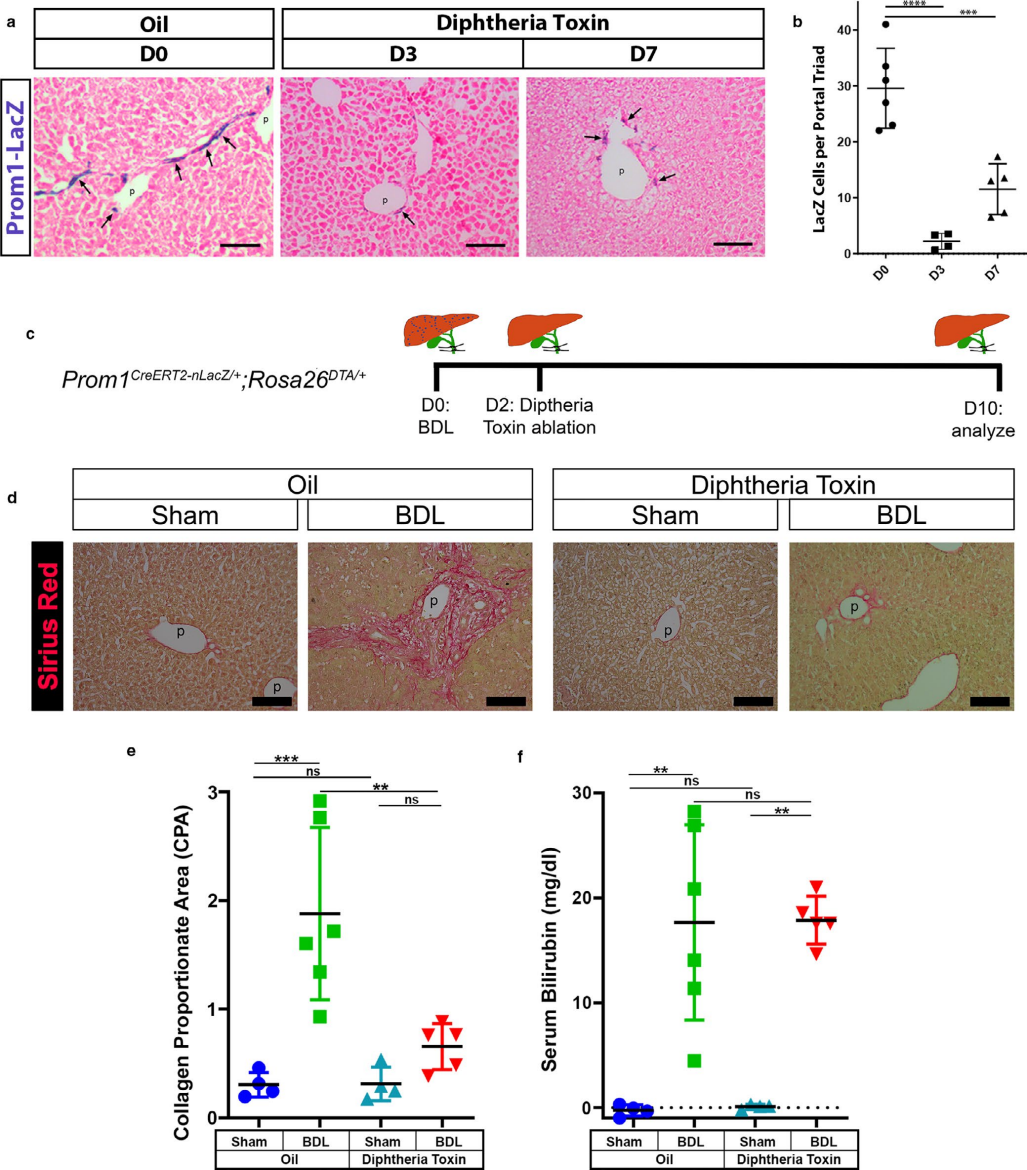
Ablation of Prom1(+) HPCs reduces biliary fibrosis induced by BDL. Six‐week‐old *Prom1^CreERT2‐nLacZ/+^;Rosa26^DTA/+^* mice were treated with tamoxifen for selective ablation of Prom1(+) HPCs via Diphtheria toxin‐induced cell death. (a) Liver tissue was analyzed on D0 (Oil, *n* = 6, two males and four female), D3 (*n* = 4, two males and two females), and D7 (*n* = 5, four males and one female) after tamoxifen injection for LacZ staining. There were no unexpected deaths prior to experimental endpoints. Arrows indicate LacZ(+) HPCs in the bile duct adjacent to the portal vein. Bar, 100 µm. (b) Quantification of LacZ cells per portal triad after ablation in A. (c) Experimental design. Six‐week‐old *Prom1^CreERT2‐nLacZ/+^;Rosa26^DTA/+^* mice were treated with tamoxifen for selective ablation of Prom1(+) HPCs 2 days after BDL or Sham operation. Corn oil was used as a negative control. The liver tissues were analyzed 10 days after surgery. Oil/Sham (*n* = 4, one male and three females), Oil/BDL (*n* = 6, four males and two females, two excluded due to early postoperative death), DT/Sham (*n* = 4, two males and two females), and DT/BDL (*n* = 5, two males and three females, one excluded for intraoperative death and two excluded for early postoperative death). (d) Sirius red staining of the liver. p, portal vein. (e) Quantification of CPA calculated from Sirius Red staining, four to eight images/animal analyzed. (f) Total serum bilirubin after sham or BDL surgery. *p < .05, **p < .01, ***p < .001, and ****p < .0001

In biliary fibrosis induced by BDL, biliary ductular reactions were associated with the development of fibrosis in the portal area. To assess the functional contribution of the Prom1(+) HPCs in response to cholestatic type injury, we depleted *Prom1*‐expressing HPCs using *Prom1^CreERT2‐nLacZ/+^;Rosa26^DTA/+^* mice, which express diphtheria toxin A in *Prom1*‐expressing cells upon tamoxifen injection (Wu et al., 2006). In order to determine the best timing for ablation, we tested for LacZ staining following tamoxifen injection. Three days after tamoxifen injection, we detected almost no LacZ staining indicating effective ablation of *Prom1*‐expressing cells in this inducible recombination model (Figure 6a,b). However, by d7, there was clear repopulation of LacZ(+) cells, suggesting repopulation of *Prom1*(+) cells.

Hence, after allowing the mice a short recovery from surgery, we opted to induce ablation of *Prom1*‐expressing cells by injection of tamoxifen (or corn oil) into *Prom1^CreERT2‐nLacZ/+^;Rosa26^DTA/+^* mice on d2 shortly after BDL or sham operation, optimally timed to coincide with proliferation of HPCs (Figure 6c). At 10 days post‐BDL, control mice injected with corn oil demonstrated marked biliary fibrosis compared to sham by collagen proportionate area (CPA) of Sirius Red staining (Figure 6d,e). In contrast, ablation of *Prom1*‐expressing HPCs resulted in significantly less portal fibrosis, similar to the control (Figure 6D,E). Serum total bilirubin was significantly elevated after BDL compared to sham controls validating the cholestatic injury of BDL in this ablation model (Figure 6f). Notably, we observed no evidence of any hepatotoxic effects of Tamoxifen in terms of CPA or serum total bilirubin levels across sham groups treated with Tamoxifen or oil controls (Figure 6e,f).

**Figure 7:**
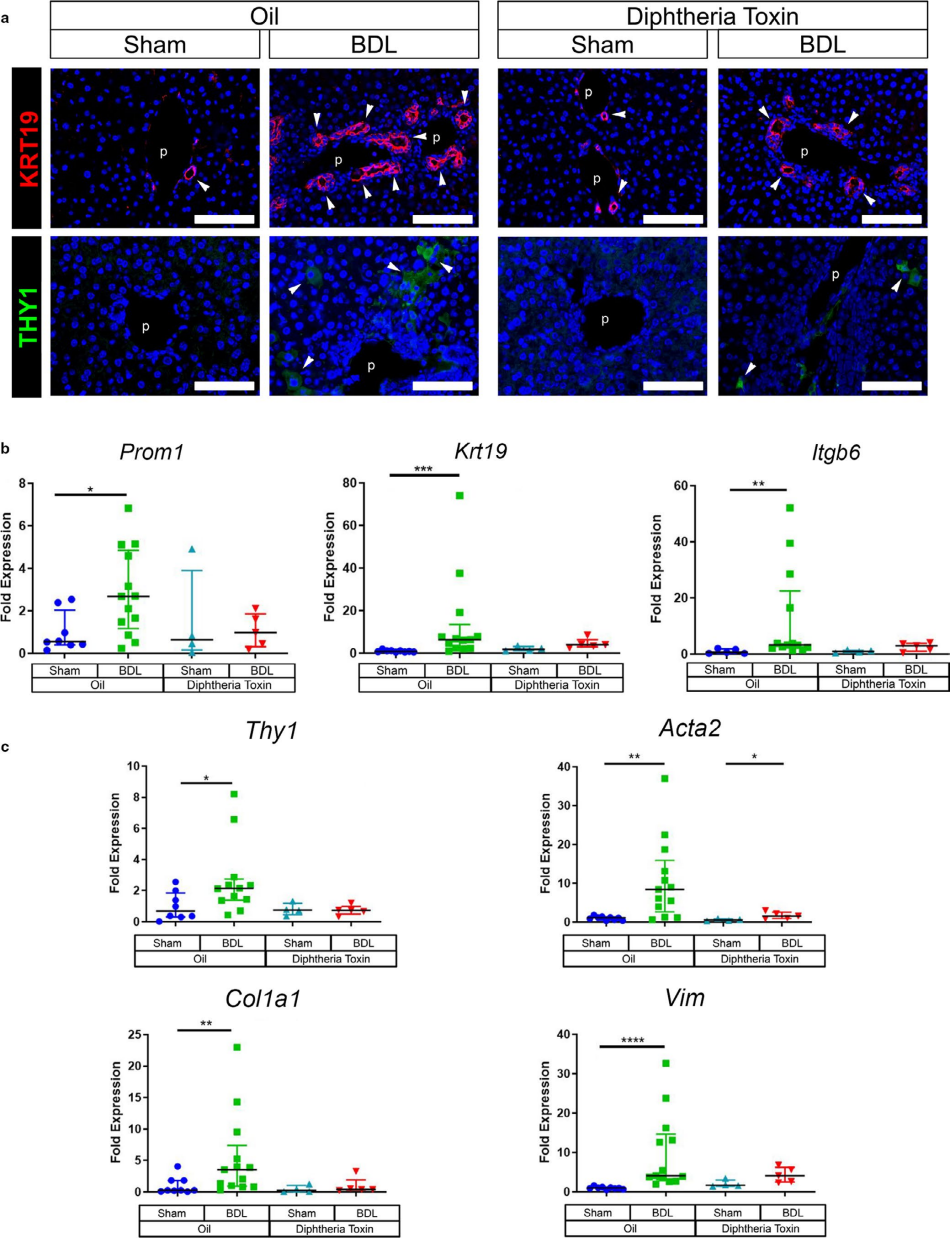
Ablation of Prom1(+) HPCs reduces fibroblast activation induced by BDL. Liver tissue prepared according to Figure 6c experimental design was analyzed with immunostaining of cholangiocytes and fibroblasts and analyzed for mRNA by qPCR of whole liver. Oil/Sham (*n* = 8, four males and four females), Oil/BDL (*n* = 12, five males and seven females, two excluded due to early postoperative death), DT/Sham (*n* = 4, two males and two females) and DT/BDL (*n* = 5, two males and three females, one excluded for intraoperative death and two excluded for early postoperative death). (a) Fluorescence immunostaining of KRT19. Arrows indicate some of the KRT19(+) cells in the bile duct adjacent to the portal vein (p). Fluorescence immunostaining of THY1. Arrows indicate THY1(+) cells, portal vein (p). Bar, 100 µm. (b) cholangiocyte markers (c) and myofibroblast markers. Each value was normalized by Gapdh. * *p* < .05, ** *p* < .01, *** *p* < .001, and **** *p* < .0001

Immunofluorescence staining of KRT19 revealed decreased biliary ductular reaction in tamoxifen‐treated mice after BDL compared to corn oil‐treated mice (Figure 7a). Moreover, immunofluorescence staining revealed a decrease in THY1(+) myofibroblasts in the biliary fibrosis by ablation of *Prom1*‐expressing HPCs after BDL compared to control (Figure 7a). We also assessed relative gene expression by QPCR of whole liver. Expression of genes encoding *Prom1*, *Krt19*, and subunit *Integrinβ6*, an activator of profibrogenic TGF‐β, was upregulated following BDL in mice treated with corn oil (Figure 7b). Following BDL in the setting of Prom1(+) HPCs ablation, the increase in expression was less albeit not statistically significant. However, we observed a significant reduction in expression of genes encoding myofibroblast markers*, Acta2, Thy1*, *Col1a1,* and *Vim* in mice after BDL in the HPC ablation group compared to control (Figure 7c). Collectively, these data indicate that the presence of *Prom1*‐expressing HPCs positively correlates with biliary fibrogenesis in cholestatic liver injury.

## 4 DISCUSSION

In this study, we demonstrated that *Prom1*‐expressing HPCs in neonatal mouse livers possess bipotential capacity to give rise to biliary epithelial cells and hepatocytes, whereas in adult livers undergoing cholestatic liver injury, they produce only biliary epithelial cells. During adult cholestatic liver injury after BDL, we observed the loss of Prom1 expression among the expansion of the *Prom1*‐expressing GFP(+) HPC lineage. RNA‐seq analysis of GFP(+) lineage demonstrated three distinct phenotypes 0, 3, and 14 days following BDL. Progeny of *Prom1‐*expressing HPCs maintained expression of biliary epithelial markers with a decrease in myofibroblastic markers and hepatocyte markers suggesting expansion of biliary lineage but not hepatocyte or myofibroblastic lineage. RNA‐seq ingenuity pathway analysis also revealed the significant downregulation of several SLRP family members known to inhibit the pro‐fibrogenic activity of TGF‐β signaling pathways with a concurrent upregulation of *Tgfb1*, *Tgfbr1*, and *Tgfbr2*. Lastly, targeted ablation of *Prom1*‐expressing HPCs during BDL with inducible Diphtheria toxin resulted in decreased fibrogenesis. Collectively, these data indicate that *Prom1*‐expressing HPCs functionally promote liver fibrosis during cholestatic liver injury.

Zhu *et al*. showed expansion of neonatal *Prom1*‐expressing HPC progeny from the periportal region ultimately comprising not only cholangiocytes but also the entire hepatic lobule of hepatocytes; in contrast, adult HPCs do not give rise to hepatocytes and appear to be quiescent (Zhu et al., 2016). Furthermore, fate tracing analyses showing GFP(+) hepatocytes throughout the hepatic lobule were interpreted to show much greater generative capacity for neonatal HPCs. In this study, we confirmed homeostatic gene expression of *Prom1* in a subset of cholangiocytes, but not in hepatocytes or mesenchymal cells in the neonatal and adult liver. Interestingly, where there is near complete GFP positivity of hepatocytes 180 days after neonatal injection, we see only a few scattered GFP(+) hepatocytes remote from portal triads at 35 days and mice injected on p42, showed no generative capacity of HPCs. The likely explanation for this is that *Prom1*‐expressing HPCs give rise to GFP(+) hepatocytes within the first few days of postnatal life but not thereafter. More likely, GFP(+) hepatocytes give rise to other GFP(+) hepatocytes to give near complete GFP positivity of hepatocytes 180 days after neonatal injection. This observation indicates a short‐lived basal capacity of HPCs to transdifferentiate into hepatocytes in the postnatal period.

Lee *et al*. demonstrated worse fibrosis following BDL in mice with *Albumin‐Cre*‐mediated hepatocyte‐specific deletion of *Prom1* (Lee et al., 2019). Whereas the authors demonstrate PROM1 immunofluorescence in hepatocytes, we and others previously observed immunostaining exclusively in hepatic progenitor cells and cholangiocytes in previous studies (Kamimoto et al., 2016; Mavila et al., 2014; Nguyen et al., 2017; Shmelkov et al., 2008; Zagory et al., 2019; Zhu et al., 2016).

In this study, we observed HPC‐specific expression of LacZ in *Prom1^CreERT2‐nLacZ^* mouse livers and recombination of GFP in *Prom1^CreERT2‐nLacZ/+^;Rosa26^Lsl‐GFP/+^* mice. In contrast to their observed SMAD7‐mediated exacerbation of BDL‐associated fibrosis in mice with embryonic *Prom1* deletion in *Albumin*‐expressing cells, here we observe decreased fibrosis with targeted ablation of *Prom1*‐expressing cells. Although we cannot exclude the possibility of weak expression of *Prom1* in hepatocytes below detection, our data, and that of others, indicate relatively stronger and specific expression of *Prom1* in hepatic progenitor cells/cholangiocytes in murine livers.

Using a variety of liver injury models, Tarlow *et al*. demonstrated that *Sox9*‐expressing HPCs give rise to cholangiocytes with either minimal or no contribution to new hepatocytes (Tarlow et al., 2014). Nguyen *et al*. showed a comparable predominance of cholangiocyte progeny derived from *Prom1*‐expressing HPCs following BDL (Nguyen et al., 2017). Kamimoto et al. (2016) reported that activation and proliferation of *Prom1*‐expressing HPCs are a stochastic and heterogeneous response to cholestatic liver injury caused by thioacetamide. Their description of continuous bands of progenitor cell lineage, which argued against migration of cells as a source of progenitor lineage, was consistent with our lineage tracing experiments. In this study, by FACS analysis, we observed a loss of Prom1 expression concurrent with expansion of the GFP(+) downstream progeny of *Prom1*‐expressing HPCs following BDL. We performed RNA‐seq analyses on GFP(+) lineage at days 0, 3, and 14 after BDL. We observed three distinct expression patterns by principal component analyses clustered by days post injury with a number of differentially expressed genes. Not surprisingly, we observed the loss of *Prom1* expression and the maintenance of expression of a number of genes encoding biliary epithelial markers and a concurrent loss of expression of genes encoding hepatocyte markers. We speculate that the transient decrease in *Epcam* expression on d3 post BDL may be due to the transition from HPC to cholangiocyte‐like cell. As *Prom1*‐expressing HPC progeny expand and differentiate under cholestatic liver injury, we see loss of hepatocyte and fibroblast markers with retention of cholangiocyte markers. These data are again consistent with predominantly, if not exclusively, biliary cell lineage of *Prom1*‐expressing HPC progeny after cholestatic liver injury. Although we demonstrate an absence of bipotential capacity in this model, *Prom1*‐expressing cells are a distinct periportal population and likely maintain progenitor cell phenotype. This is supported by our RNA‐seq data in which at d0, we show expression of markers for hepatocytes, cholangiocytes, and fibroblasts. Additionally, there is evidence to suggest that under certain conditions, HPCs can give rise to a small number of hepatocytes during cholestatic liver disease and hepatotoxic liver disease, such as in Espanol‐Suner et al. (2012) and Tarlow et al. (2014).

We recently reported that *Prom1* knockout mice develop less ductular reaction and fibrosis in the mouse Rhesus rotavirus‐mediated BA model (Zagory et al., 2019). To determine whether *Prom1*‐expressing HPCs play a role in biliary fibrosis, we ablated them using *Prom1^CreERT2‐nLacZ/+^;Rosa26^DTA/+^* mice. We observed less ductular reaction and biliary fibrosis in the DTA ablation of *Prom1*‐expressing cells after BDL. THY1(+) portal fibroblast expansion, which is histologically evident after BDL with upregulation of portal fibroblast mRNA markers, is also significantly reduced following *Prom1*‐expressing cell ablation. Given our RNA‐seq cell lineage data, *Prom1*‐expressing HPCs GFP(+) progeny do not appear to be the source of collagen production nor do they appear to differentiate into fibroblast‐like cells. Given these data collectively, we posit that Prom1(+) HPCs and their progeny may contribute to the activation of portal fibroblasts and, hence, biliary fibrosis. It has previously been reported that during early cholestatic liver fibrosis, type 1 Collagen is primarily produced via myofibroblasts derived from activated portal fibroblasts (Iwaisako et al., 2014). TGF‐β1 is known to induce myofibroblasts from portal fibroblasts and hepatic stellate cells in liver fibrosis (Lua et al., 2016). Integrin‐αvβ6 is expressed in ductular reactions (Peng et al., 2016). A unique function of this integrin dimer is that it binds to the latent form of TGF‐β1 and activates it on epithelial cells (Annes, Chen, Munger, & Rifkin, 2004). The expression of *Itgβ6*, which encodes one of the subunits consisting of Integrin αvβ6, is upregulated in the fibrotic liver after BDL. After ablation of Prom1(+) HPCs, its expression was not induced by BDL. We do not specifically observe a change in relative expression of *Itgβ6* within our GFP (+) HPC lineage population after BDL. However, we do observe a reduction in ductular reactions and expression of *Itgβ6* following ablation of Prom1‐expressing cells. We speculate based on our data that *Prom1*‐expressing HPCs play an important role likely via downstream cholangiocyte‐mediated activation of portal fibroblasts possibly through regulation of the TGF‐β1 pathway, contributing to the fibrogenesis of cholestatic liver injury. Further studies are necessary to more definitively define Prom1(+) HPCs influence on portal fibroblasts in the fibrogenesis of cholestatic liver disease.

Possible links between *Prom1* and portal fibroblast activation are identified within our RNA‐seq data. Evaluation of the most differently expressed genes after BDL revealed significant downregulation of several members of the SLRP family. The SLRP family is known to suppress the activity of TGF‐β signaling pathways (Costanza et al., 2017). Dcn inhibits TGF‐β activity at multiple locations along the pathway including high affinity binding to all TGF‐β isoforms, inhibition of phosphorylation of SMAD2, cytoplasmic sequestration of SMAD4, and decreased synthesis of TGF‐β1 and TGF‐β2 (Costanza et al., 2017). Several studies have demonstrated that endogenous Dcn inhibits TGF‐β1 activation and reduced fibrosis in mouse lung and diabetic nephropathy models, as well as Dcn‐deficient models aggravated liver and kidney fibrosis via increased TGF‐β activation (Baghy et al., 2011; Kolb et al., 2001; Merline et al., 2009; Williams et al., 2007). Bgn and Dcn reduce TGF‐β activity in the context of lung fibrosis (Kolb et al., 2001). Extracellular matrix Lum is an endogenous inhibitor of TGF‐β2 activity, as well as its downstream regulators, SMAD2, integrin β1, and FAK (Nikitovic et al., 2011). *Lum*‐null mice exhibit increased activation of HSCs (Krishnan et al., 2012). Aspn inhibits this pathway by direct binding of TGF‐β1 (Maris et al., 2015). Our RNA‐seq ingenuity pathway analysis data demonstrate strong downregulation of these SLRP‐related genes as well as upregulation of *Tgfbr1*, *Tgfbr2*, all consistent with TGF‐β signaling pathway activation. The SLRP family may be a potential link between Prom1(+) HPCs and the activation of portal fibroblasts in biliary fibrosis and will be an area of future study.

Interestingly, Prom1(+) progeny upregulates expression of *Mmp7*, which is a marker for BA ( Lertudomphonwanit et al., 2017). Recent studies using single cell analysis highlight essential role of YAP signaling in ductular reaction (Pepe‐Mooney et al., 2019; Planas‐Paz et al., 2019). Interestingly, we failed to detect the expression of YAP target genes, such as *Ctgf* and *Cyr61*, in Prom1(+) cells by RNA‐seq analysis, implying their unique character and role in biliary fibrosis.

In summary, *Prom1*‐expressing HPCs give rise to cholangiocytes comprising ductular reactions during cholestatic injury following bile duct ligation within regions of developing fibrosis. Progeny derived from Prom1‐expressing HPCs expresses genes integral to TGF‐β pathway activation and loss of Prom1‐expressing HPCs is associated with decreased fibrosis. Focusing on the role Prom1 plays during liver fibrogenesis may provide insight into potential therapeutic interventions in patients with cholestatic liver injury.

## 5 GEO Accession number for RNA‐seq data

GSE144726 (reviewer access token: ejshosyonlkznif).

## CONFLICT OF INTEREST

None declared.

## AUTHOR CONTRIBUTIONS

M.F., K.A., and K.W. involved in the conception and design of research; M.F., J.X., C.S., N.M., E.M., M.H., A.G., and C.L. performed experiments; M.F. and C.S. analyzed data; M.F., C.S., K.A., and K.W. interpreted the results of experiments; M.F., C.S., A.G., and K.W. prepared figures; M.F., C.S., K.A., and K.W. drafted manuscript.
